# Migration and Chondrogenesis of Cells from Minced Nasal Cartilage in Type I Collagen Hydrogel: A Workflow for One-Step Engineering of Injectable Grafts

**DOI:** 10.3390/gels12030190

**Published:** 2026-02-25

**Authors:** Alexander Gensch, Atharva Damle, Orhan Sonsöz, Diana Mock, Martin Haug, Davide Adamo, Ewelina M. Bartoszek, Gyözö Lehoczky, Ivan Martin, Andrea Barbero

**Affiliations:** 1 Department of Biomedicine, University Hospital Basel, University of Basel, 4031 Basel, Switzerlandatharva.damle@unibas.ch (A.D.); gyoezoe.lehoczky@ukbb.ch (G.L.); ivan.martin@usb.ch (I.M.); 2 Plastic, Reconstructive, Aesthetic, and Hand Surgery, University Hospital Basel, 4031 Basel, Switzerland; 3 Center for Regenerative Medicine “Stefano Ferrari”, University of Modena and Reggio Emilia, 41125 Modena, Italy; davide.adamo@unimore.it; 4 Department of Life Sciences, University of Modena and Reggio Emilia, 41125 Modena, Italy; 5 Microscopy Core Facility, Department of Biomedicine, University of Basel, 4031 Basel, Switzerland; 6 University Children’s Hospital Basel, 4031 Basel, Switzerland; 7 Orthopaedic Surgery and Traumatology, University Hospital Basel, 4031 Basel, Switzerland

**Keywords:** cartilage repair, minced cartilage, nasal chondrocytes

## Abstract

Articular cartilage (AC) damage heals poorly and can progress to osteoarthritis. Implantation of AC fragments (Minced Cartilage Implantation, MCI) is a promising one-step repair technique but is constrained by the limited availability of healthy AC. In this study, we evaluated the feasibility of MCI using nasal septal cartilage (NSC) as an alternative source of hyaline tissue with strong regenerative capacity. NSC obtained from rhinoplasties was decontaminated using a novel protocol, minced with or without Poloxamer 188 (P188), embedded in collagen I gel (0.5 mL per sample), and cultured for 42 days in platelet-rich plasma (PRP)-supplemented medium. The decontamination procedure with a combination of antibiotics was effective and did not impair cell viability. Histology of the resulting constructs confirmed robust cellular outgrowth and matrix deposition. Tissues produced from NSC and fragmented with P188 contained more cartilaginous matrix than those from NSC fragmented without P188 and those from AC fragmented with P188. NSC fragments embedded in a 1 mL hydrogel, sufficient for clinically relevant defect volumes, also demonstrated strong outgrowth and satisfactory matrix formation. Overall, the developed protocol supports the use of NSC as a viable tissue source in gel-based, injectable MCI grafts for focal cartilage repair.

## 1. Introduction

Focal cartilage defects present a persistent clinical challenge due to the limited regenerative capacity of cartilage tissue. The poor regenerative capacity of cartilage stems primarily from its avascular nature and its low cellular turnover [1,2]. Current surgical treatments such as autologous chondrocyte implantation (ACI) are constrained by high treatment costs, and the need for multiple surgeries [3]. Furthermore, ACI is associated with donor-site morbidity resulting from the harvesting of healthy cartilage [4] and the variable regenerative capacity of expanded articular chondrocytes [5]. A major contributor to this variability is the tendency of articular chondrocytes to de-differentiate during in vitro expansion, leading to the acquisition of a fibroblast-like phenotype characterized by a reduced expression of cartilage-specific matrix components such as collagen type II and aggrecan [6,7,8]. Emerging one-step techniques, which eliminate the need for cell culture steps, like minced cartilage implantation (MCI) offer some promise [9]. Cartilage for MCI is generally harvested directly from the debridement of the defect. Although this approach is cost-effective and avoids collecting cartilage from healthy donor sites, it is plausible that cartilage from the lesion area may already be structurally compromised or affected by inflammatory changes following joint trauma, which could limit regenerative performance [10]. Additionally, donor-dependency in the performance of articular chondrocytes remains a concern [11]. These limitations underscore the need for an alternative source of cartilage tissue for MCI applications.

Nasal septal cartilage (NSC) represents an autologous alternative for cartilage tissue-engineering therapies, combining hyaline-like characteristics, similar to articular cartilage, with minimally invasive harvestability [12]. In particular, nasal chondrocytes (NCs) have shown a higher proliferative and differentiation potential in addition to their superior regenerative capacity, compared to articular chondrocytes, partially attributed to their neuro-ectodermal origin [13,14,15]. Moreover, NCs possess remarkable plasticity, enabling them to integrate into articular cartilage [16,17]. Based on these findings, cartilage grafts engineered by NCs were used to repair knee cartilage defects, with excellent clinical outcomes [18,19]. Recently, we also showed that rapidly isolated NCs sequentially embedded in a hydrogel can efficiently proliferate and produce hyaline cartilage tissue in vitro and in vivo [20].

Building on these findings, the direct use of mechanically fragmented NSC, without prior cell expansion, could offer significant procedural and economic advantages. However, mechanical fragmentation exposes chondrocytes to shear forces, leading to cell damage and apoptosis [21,22]. The application of a protective additive may counteract these mechanical stresses and thereby improve the viability and performance of cells within the cartilage fragments. In this context, the amphiphilic surfactant Poloxamer 188 (P188) has shown promising cytoprotective effects in various models of joint injury. P188 helps preserve chondrocyte viability by stabilizing cell membranes, preventing calcium overload, and reducing apoptosis and inflammation caused by mechanical stress [23,24]. Intra-articular administration of P188 has been demonstrated to reduce cell death and preserve extracellular matrix integrity following blunt trauma to meniscal and articular cartilage tissues in several preclinical studies [25,26,27]. Despite these encouraging results, the use of P188 during the cartilage mincing process remains unexplored. We therefore hypothesize that the application of P188 may counteract cartilage mincing-mediated stress, thereby enhancing the viability and regenerative performance of cells within the cartilage fragments.

To ensure the safety of nasal cartilage transplantation, it is paramount to address the risk of microbial contamination, as the nasal cavity hosts diverse microbiota. Transplantation of contaminated tissue into a sterile joint environment may lead to graft failure or serious infection. Therefore, a reliable and non-destructive decontamination protocol is crucial for clinical translation [28].

A further challenge lies in stabilizing the minced cartilage fragments within the defect site. While resorbable membranes or fibrin glue are commonly used [29], injectable hydrogels offer an alternative with superior adaptability and biocompatibility.

The overarching goal of this study is to develop and evaluate a clinically translatable method for the treatment of focal cartilage defects using mechanically minced nasal cartilage embedded in a collagen-based hydrogel. To support this objective, we conducted a series of in vitro experiments addressing the following questions:

1. Can NSC be reliably decontaminated using an antibiotic washing solution without compromising cell viability or outgrowth?
2. Can chondrocytes migrate out of the fragmented NSC, colonize the surrounding collagen I gel, and initiate the production of hyaline-like chondrogenic matrix?
3. Does supplementation of P188 during the mechanical fragmentation of the NSC enhance cell viability, migration/colonization, and biosynthetic performance?

Our results demonstrated effective NSC decontamination without detrimental effects on chondrocyte outgrowth or the capacity of the cells growing out of the fragments to produce cartilaginous matrix within the collagen gel. Furthermore, we showed that P188 enhanced the quality of the newly formed cartilaginous tissue. These findings provide robust preclinical evidence for the therapeutic potential of this approach.

## 2. Results and Discussion

### 2.1. Biopsy Decontamination

To examine whether a brief antibiotic wash affects nasal septal cartilage (NSC), beyond achieving decontamination, we used a paired, donor-matched design comparing phosphate-buffered saline (PBS)-washed untreated biopsies (UT) with antibiotic-treated biopsies (TR).

Outcomes of microbial outcomes summarized in Table 1 show that, as expected, the transport medium (TM) samples collected from NSC specimens exposed to the patient’s own nasal mucosa were positive in the BacT/ALERT system (5/5). In contrast, samples washed with PBS alone (untreated (UT)) and those washed with the decontamination solution (DS) (containing antibiotics, treated (TR)) yielded negative results in BacT/ALERT (0/5 each), demonstrating reliable decontamination. Although PBS washes alone produced negative results under laboratory settings, clinical application should prioritize an antibiotic-augmented decontamination solution to ensure patient safety, provided the exposure is brief and remains within non-toxic thresholds. Consistent with this, the absence of cytotoxicity following antibiotic treatment resulted in no significant difference between UT and TR groups.

Chondrocyte viability measured at day 0 was comparable (less than 10% difference in the live cells (%)) between UT and TR (ns), indicating no acute cytotoxicity mediated by ~20 min exposure of the cartilage specimens to the antibiotics. Notably, dose- and time-dependent toxicity has been reported for prolonged exposure to certain antibiotics or high concentrations [30,31], reinforcing the rationale for utilizing brief, low-dose washes.

We next evaluated whether treating NSC with DS could negatively affect cell growth from the fragments, either when placed in well plates or embedded in type-I collagen gel (ChondroFiller^®^, meidrix biomedicals GmbH, Esslingen, Germany).

**Figure 1:**
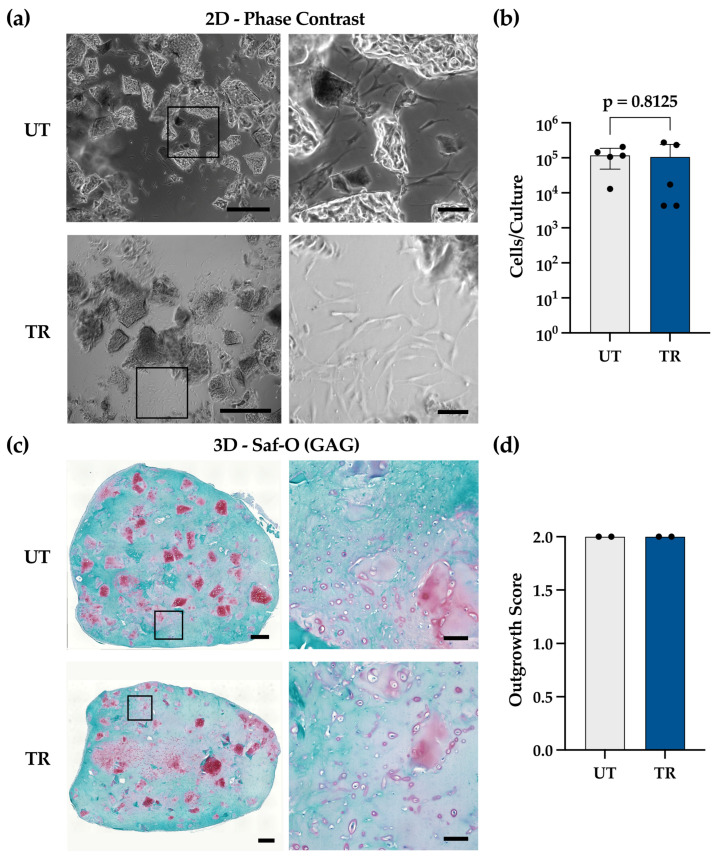
(**a**) Phase-contrast microscopic images of 2D-cultured fragments. Scale bars: overviews 500 µm, zooms 100 µm. Black squares in the left panels indicate regions shown in higher magnification in the corresponding right panels; (**b**) Quantification of phase-contrast images of 2D cell outgrowth (total cells per culture, incl. cells in chip fragments). Donor-paired statistical analysis was performed using Wilcoxon matched-pairs signed-rank test, two-tailed, *p* = 0.8125; *N* = 5 donors; 3 wells/group/donor: comparable across conditions. (**c**) Brightfield images of Safranin-O (Saf-O)-stained slides of 3D-cultured cartilage chips in both conditions. Scale bars: overviews 500 µm, zooms 100 µm. Black squares in the left panels indicate regions shown in higher magnification in the corresponding right panels (**d**) Estimation of 3D cell colonization (cells in gel; chip-remnant cells excluded): comparable across conditions (*N* = 2 donors; 1 construct/donor) (mean score of 6 predefined section levels per construct). Donor-paired statistical analysis could not be performed due to small *N* size.

Phase-contrast images of 2D-cultured NSC fragments at 28 days (Figure 1a) showed similarly pronounced cellular outgrowth from the cartilage chips in both conditions (UT and TR). DNA quantification (Figure 1b) confirmed comparable amounts of cells colonizing the wells in UT and TR groups. Safranin-O/Fast Green images of 3D constructs generated by embedding and culturing NSC fragments in Collagen I gels also indicated similar cellularity in both groups (Figure 1c). Moreover, cell quantification confirmed that the extent of gel colonization was comparable between the UT and TR groups (Figure 1d).

These findings support the potential use of NSC biopsies directly in regenerative applications without compromising patient safety. Our results align with prior reports demonstrating that brief antibiotic rinsing can decontaminate nasal cartilage without compromising subsequent cell culture [30,32]. Furthermore, our results demonstrate the robust capacity of NCs to migrate from the fragments and proliferate independently of antibiotics treatment, regardless of culture conditions.

### 2.2. P188 Enhances the Performance of Minced NSC in Collagen I Gel

Poloxamer 188 (P188) has previously been shown to preserve chondrocyte viability following mechanical stresses of articular cartilage and meniscal tissue [24,33]. We therefore investigated whether P188 could exert a similar protective effect on nasal chondrocytes (NCs). For this pilot experiment, nasal septal cartilage (NSC) from three donors was minced in the absence (Ctr) or presence of Poloxamer 188 (P188) and incubated for 1 h. Following an additional 24 h of culture in complete medium for viability (CM-V), fragments were digested with type II collagenase, and cell viability was determined by Trypan blue exclusion assay. As shown in Figure S1, P188 treatment increased cell viability in all the donors (though to a varying extent) compared to the matched controls.

**Figure 2:**
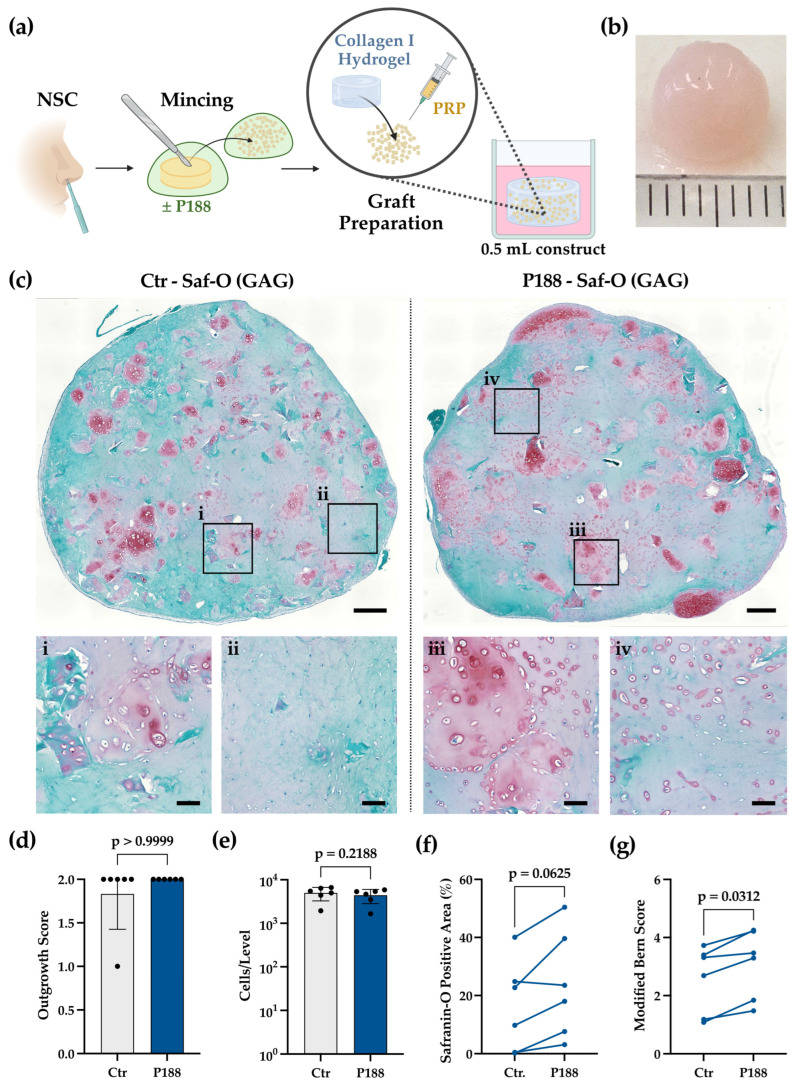
(**a**) Schematic representation of the experimental workflow for nasal septal cartilage (NSC) mincing and graft preparation. (**b**) Macroscopic view of a representative construct (P188 group) after 42 days of culture. (**c**) Safranin-O (Saf-O) staining images showing “best” donor-paired outcomes for control (Ctr, (**left**)) and P188 (**right**) groups. Black squares in the top panels (**i**–**iv**) indicate regions shown in higher magnification in the bottom panels. Scale bars: overviews 500 µm, zooms 100 µm. (**d**–**g**) Donor-paired statistical analysis was performed using Wilcoxon matched-pairs signed-rank test, two-tailed, (*N* = 4 donors; 1–2 constructs/donor), based on the mean of six predefined section levels per construct. (**d**) Outgrowth score (*p* > 0.9999). (**e**) Total cell number per section level (*p* = 0.2188). (**f**) Safranin-O (Saf-O) positive matrix area (*p* = 0.0625); and (**g**) Modified Bern Score (*p* = 0.0312).

We subsequently assessed whether P188 enhances the performance of NCs following NSC mincing. Samples were chopped in the presence or absence of P188, and resulting fragments were cultured in type-I collagen gel (ChondroFiller^®^) for 42 days (Figure 2a).

During the course of the 42-day culture period, Ctr as well as P188 constructs compacted in a similar fashion by approximately 30% in diameter (from ~10 mm to ~7 mm) and appeared visibly opaque as represented in Figure 2b. The constructs exhibited a cohesive, hemispherical shape, with a smooth, glossy surface and uniform thickness in both groups. Safranin-O/Fast Green staining of P188-treated constructs revealed uniform and intense red-stained regions, indicating evenly distributed sulfated glycosaminoglycans (sGAG) deposition, with rounded chondrocytes situated in well-defined lacunae (Figure 2c). In contrast, Ctr constructs often displayed patchy staining, and fibrocellular regions containing cells with elongated morphology (Figure 2c). P188 constructs retained focal zones of rounded morphology (Figure 2c), suggesting greater phenotypic stability and increased matrix-deposition even in the lowest-performing donors (Figure S2).

Qualitative outgrowth scoring (Figure 2d) revealed that P188 did not further increase cell outgrowth compared to Ctr. Similarly, quantitative assessment of total cell number per construct (Figure 2e) showed no significant difference between Ctr and P188 (*p* = 0.2188), suggesting that P188 may not directly stimulate cell migration or proliferation. However, a strong trend towards more extensive Safranin-O-stained regions was observed in the P188 constructs (*p* = 0.0625) (Figure 2f), indicating enhanced proteoglycan deposition. The Modified Bern Score (MBS), which integrates semi-quantitative evaluation of Safranin-O-staining intensity and cell morphology, was higher in every P188 construct compared to its matched control, demonstrating superior cartilaginous quality in this group (*p* = 0.0312) (Figure 2g). Separate analysis of the two MBS sub-categories (Table 2) confirmed that P188 treatment significantly enhances both, GAG deposition and the maintenance of chondrocytic phenotype.

The superior outcomes observed after P188 treatment can be traced back to its ability to confer early membrane protection, which preserves cellular integrity and helps cells withstand the stress induced by the scalpel during the cartilage-mincing step. Prior work shows that P188 reduces chondrocyte death after blunt joint injury in vivo [34], decreases DNA fragmentation and apoptosis in chondrocytes following impact loading of bovine articular cartilage explants [33], and maintains viability while impeding apoptotic signaling in human cartilage under acute compression [24]. These established mechanisms provide a plausible rationale for the present findings. Nevertheless, to fully elucidate the mechanistic action of P188 as a protective agent that imparts membrane stabilization, a comprehensive study involving multiple donors is imperative. Taken together, although P188 does not alter cell numbers, it enhances NC phenotypic preservation and biosynthetic activity within a clinically relevant collagen I environment.

### 2.3. Immunohistochemical Characterization of NSC Constructs

To further examine the composition of the tissue generated by the NSC fragments in the collagen I gel, and to investigate the mechanisms underlying the histological improvements observed in the P188 group, immunohistochemical characterization was performed on construct sections from the matched-donor cohort.

**Figure 3:**
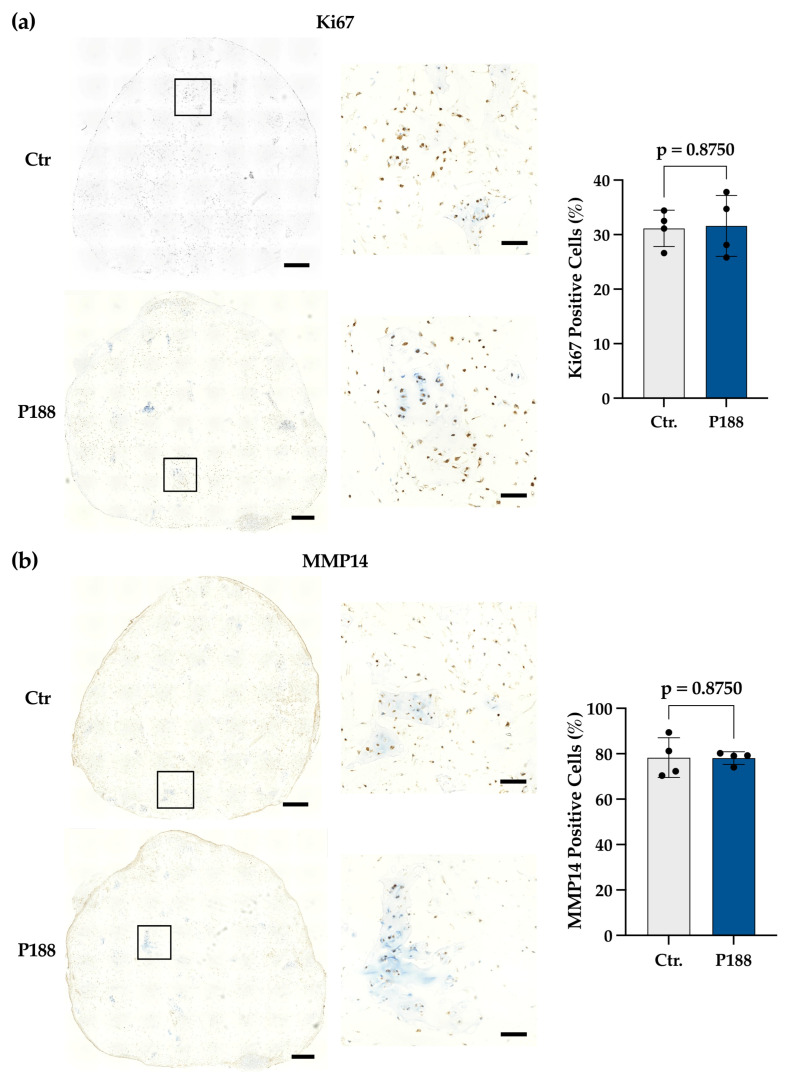

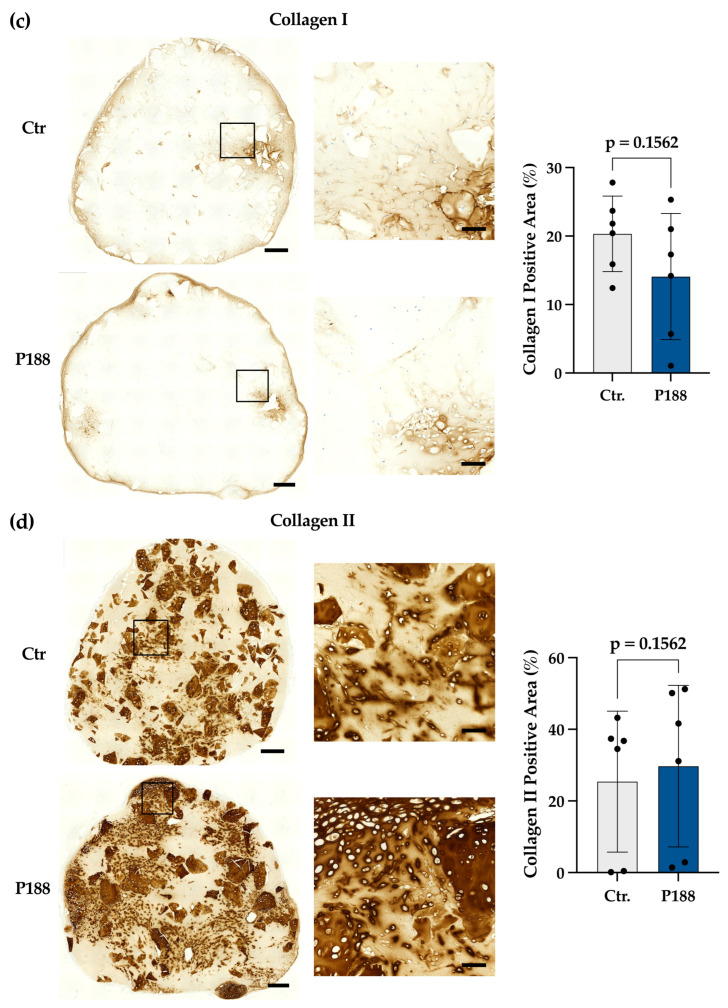
Immunohistochemical stainings for ’best’ performing constructs (**a**) Ki-67, (**b**) MMP14 (MT1-MMP), (**c**) Collagen I, and (**d**) Collagen II in Ctr and P188 constructs. Scale bars: 500 µm overviews, zooms 100 µm. Black squares in the left panels indicate regions shown in higher magnification in the corresponding right panels. Quantitative analysis was performed in QuPath (v0.5.0) based on the mean of six predefined section levels per construct. Donor-paired statistical analysis was performed using Wilcoxon matched-pairs signed-rank test, two-tailed, Ki-67 and MMP14, *N* = 2 donors (2 constructs/donor) col I and col II, *N* = 4 donors (1–2 constructs/donor). *p* values: Ki-67 = 0.8750, MMP14 = 0.8750, Collagen I = 0.1562, Collagen II = 0.1562.

In these preliminary observations (*N* = 2), the proportion of Ki-67-positive nuclei (Figure 3a) was comparable between Ctr and P188 constructs, suggesting that P188 may not exert a sustained pro-proliferative effect. Similarly, exploratory analysis showed that MMP14 (MT1-MMP), a marker for tissue remodeling, was expressed to a similar extent in both groups (Figure 3b), particularly at construct rims and chip-gel interfaces. This stable expression is consistent with the ‘fragmentation effect’ previously observed in an in vitro articular cartilage model migration [35]. In that study, Lei et al. observed that cartilage fragmentation upregulates MMP14 expression and that this catabolic factor is required for chondrocyte to egress from tissue fragments [35]; in turn, its overexpression enhances migration. The finding that P188 did not alter MMP14 expression here suggests that its effect may not be MMP-mediated, but rather driven by mechanical or structural membrane stabilization, as current evidence suggests [24,33,34].

Figure 3c shows that the expression of collagen type I (col I), a fibrocartilage-associated matrix protein, is predominantly localized to the construct periphery, with sparse intragel-staining associated with immature cartilage formation. A modest but non-significant reduction in positive area was observed in the P188 group, where the intragel staining was associated with lacunar chondrocytes indicative of mature cartilage formation (*p* = 0.1562). In contrast, collagen type II (col II) deposition (Figure 3d) was abundant in both groups. Nevertheless, a trend towards an increment in the col II-positive area was observed in the P188 group (*p* = 0.1562). Collectively, these findings suggest a favorable shift toward a more hyaline-like matrix phenotype in the P188 group, characterized by reduced col I expression and abundant col II matrix generation. This interpretation is consistent with early findings by Shakibaei et al., showing that col I associates with α1-integrin during early, undifferentiated stages and declines as differentiation proceeds, whereas col II levels increase alongside α3-integrin during terminal chondrocyte differentiation [36]. Together, this evidence suggests that P188 application during cartilage mincing can foster stable chondrogenic maturation within the gel. Nevertheless, an in-depth transcriptomic or biochemical analysis is required to elucidate the key genes regulating P188-induced cytoprotection.

### 2.4. Performance of Minced Articular Cartilage in Collagen I Gel

**Figure 4:**
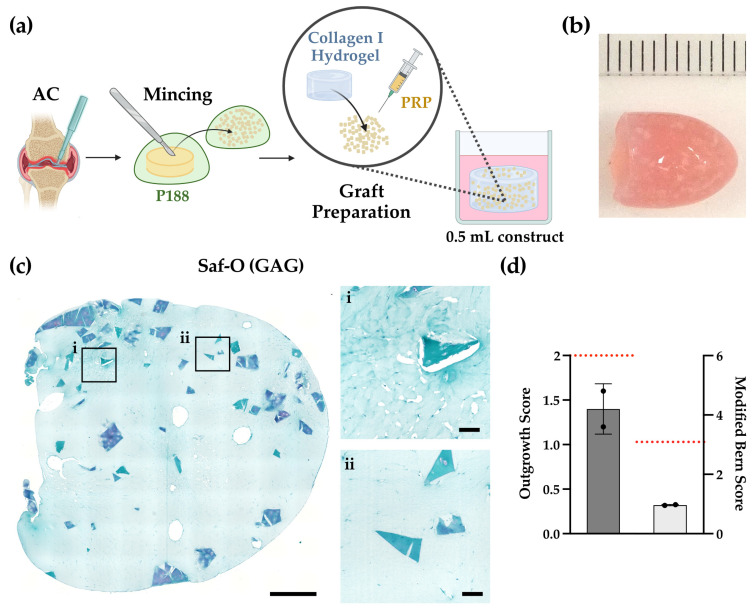
(**a**) Schematic representation of the experimental workflow for articular cartilage (AC) mincing (in the presence of P188) and graft preparation (**b**) Representative macroscopic view of minced articular cartilage (MAC) constructs generated in a collagen I gel cultured for 42 days (to be compared with that in Figure 2b. (**c**) Safranin-O (Saf-O) staining of representative images. Scale bars: overviews 500 µm, zooms 100 µm. Black squares in the left panel (**i**,**ii**) indicate regions shown in higher magnification in the right panels; (**d**) Outgrowth score ((**left**) *y*-axis); Modified Bern Score ((**right**) *y*-axis), *N* = 2 donors, 5 slides/donor; red dash lines represent mean values of constructs generated with minced nasal septal cartilage (MNC).

To evaluate how articular cartilage compares to minced NSC, we assessed minced articular cartilage (AC) from osteoarthritic (OA) patients (Figure 4a). For harvesting and subsequent mincing, only macroscopically intact regions were identified. Although an OA background implies potential exposure to low-grade inflammation and catabolic signaling, these donor characteristics reflect the pathological reality of knee defects; minced articular cartilage implantation typically employs perilesional or debrided tissue that may also carry inflammatory or degenerative hallmarks.

Unlike minced nasal septal cartilage (MNC) constructs (Figure 2b), those generated with minced articular cartilage (MAC, *N* = 2 donors) appeared translucent with clearly visible cartilage fragments and exhibited minimal compaction over the culture period (from ~10 mm to ~9 mm) (Figure 4b). This reduced compaction and translucent appearance likely indicate lower cellular remodeling activity and limited deposition of new cartilaginous matrix. Microscopic evaluation (Figure 4c) revealed sparsely distributed cells with negligible Safranin-O staining, confirming impaired chondrogenic matrix formation. The outgrown cells were predominantly fibroblast-like, arranged in loose, non-lacunar clusters, with no evidence of rounded, hyaline-like organization.

Quantitative outgrowth analysis (Figure 4d) further highlighted this limited performance. Although all MAC constructs showed consistent outgrowth, the scores remained moderate (1.4 ± 0.5). When directly compared to the MNC (red dashed reference line), the reduced outgrowth of cells from MAC became particularly evident, as all MAC samples remained well below the MNC average. These data confirm that while articular chondrocytes retain migratory competence, it occurs at a significantly reduced magnitude compared to nasal chondrocytes. Similarly, the Modified Bern Score (Figure 4d) showed markedly lower values for MAC compared with the MNC constructs (red reference line), underscoring the limited matrix-forming capacity of OA-derived cells under these conditions.

Under identical conditions optimized for MNC, articular cartilage exhibited modest outgrowth without hyaline-like matrix deposition. The fibroblastic morphology of the cells colonizing the gel implies the persistence of a repair-oriented phenotype, potentially stemming from residual inflammatory priming common in OA tissue. These preliminary observations align with Levinson et al., who observed cell migration but no proteoglycan-rich matrix in MAC embedded in collagen/PRP gels [21].

In summary, as a feasibility observation, under culture conditions optimized for MNC, MAC from OA donors showed moderate but consistent outgrowth without hyaline-like matrix formation. This indicates that while migratory capacity is preserved, the regenerative potential remains limited, likely due to the inflammatory and degenerative background of OA tissue. This limited performance of MAC may be attributed to donor age, in addition to the tissue’s osteoarthritic origin.

Given that cartilage tissues for MAC implantation are typically harvested from non-osteoarthritic joints, future experiments should utilize cartilage fragments derived from both the nasal cartilage and focal articular cartilage defects from the same donor to substantiate the performance of MNC over MAC. Our previous results, demonstrating the superior cartilage-forming capacity of matched NCs compared to debrided knee chondrocytes (harvested from patients with focal cartilage defects) [37], suggest that the implantation of MNC may provide a superior alternative to MAC for effective cartilage repair.

### 2.5. Performance of Clinically Relevant Constructs

To reflect the dimensional characteristics of clinically used cartilage repair constructs, we selected a 1 mL collagen model as a clinically relevant implant volume. This scale corresponds to the typical range of focal cartilage defect coverage in current regenerative therapies, which usually treat lesion sizes of approximately 2–6 cm^2^ with a tissue thickness of 1–2 mm. Such dimensions yield a total implant volume of about 0.2–1.2 mL, aligning closely with the size range reported for cartilage grafts used in clinical studies [38,39,40]. The 1 mL construct was thus chosen as a clinically meaningful and experimentally scalable reference format for the subsequent in vitro investigations.

**Figure 5:**
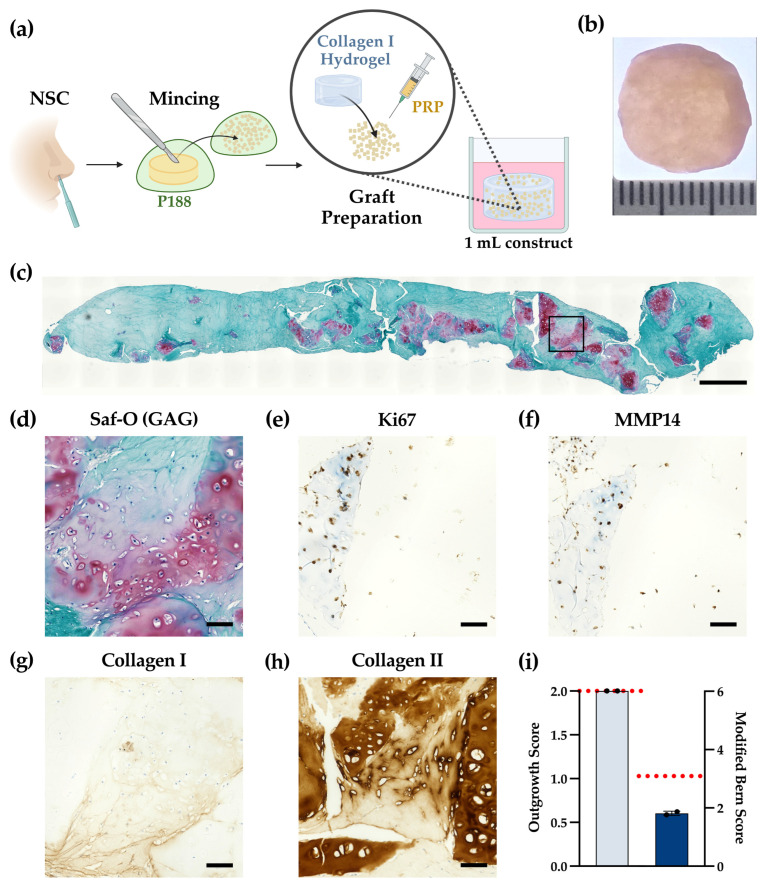
(**a**) Schematic representation of experimental workflow for nasal septal cartilage (NSC) mincing and preparation of the clinically relevant 1 mL construct. (**b**) Representative macroscopic view of a representative construct after 42 days of culture. (**c**) Safranin-O (Saf-O) overview showing proteoglycan-rich cartilaginous islands within the collagen gel. Scale bar: 1 mm. Black square in the top panel indicates the region shown in higher magnification in the bottom panels (**d**–**h**) High-magnification images. Scale bars: 100 µm. (**e**) Ki-67 immunostaining indicating numerous proliferating cells. (**f**) MMP14 immunostaining showing matrix remodeling at the construct periphery, within the gel and in cartilage chips. (**g**) Collagen I immunostaining predominantly in the surrounding gel and pericellular strands. (**h**) Collagen II immunostaining within cartilaginous islands and outgrowth zones. (**i**) Outgrowth score ((**left**) *y*-axis) and Modified Bern Score ((**right**) *y*-axis) for the representative 1 mL construct, *N* = 1 donor, 6 slides/donor; the red dashed line indicates the mean value of the 0.5 mL constructs.

Cartilage fragments, derived by chopping 6 mm NSC biopsies (corresponding to 60–80 mg) from two donors in the presence of P188, were embedded and cultured in 1 mL collagen I gel for 42 days (Figure 5a), as previously described. The resulting constructs appeared smooth, disk-like, and cohesive, with no fissures or necrotic regions (Figure 5b). Surface homogeneity was comparable to smaller-scale constructs generated previously, although, histologically, they appeared overall less cartilaginous.

Safranin-O/Fast Green staining (Figure 5c,d) revealed proteoglycan-rich islands that formed primarily around the chip clusters and extended into the collagen gel. In contrast, the inter-island regions were lightly or negatively stained for Safranin-O. High-magnification views confirmed rounded, lacuna-bearing chondrocytes and territorial matrix localized at the outgrowth fronts, with cells distributed throughout the gel. Ki-67 immunostaining (Figure 5e) showed numerous positive nuclei, indicating robust proliferative activity on day 42. MMP14 immunostaining (Figure 5f) was readily detectable at the construct periphery, throughout the collagen gel, and within the cartilage chips, with focal-to-diffuse pericellular and interstitial signals, consistent with active-matrix remodeling at this time point. Col II immunostaining (Figure 5h) was pronounced within cartilaginous islands and outgrowth zones, while col I (Figure 5g) localized mainly to the surrounding gel and pericellular strands. Safranin-O-positive regions spatially co-localized with col II across examined sections, supporting hyaline-like matrix deposition proximal to chip-derived outgrowth areas and a more fibro-like background in the bulk gel.

The results of the scorings in Figure 5i show that the clinically relevant constructs achieved a high outgrowth score, within the range of the previously generated smaller-scale constructs, although they exhibited slightly lower MBS values. Overall, these findings primarily demonstrate the feasibility of generating a clinically relevant 1 mL construct; however, they do not provide sufficient evidence of a significant difference in tissue quality compared to the 0.5 mL constructs. Furthermore, these findings should be interpreted with caution given the limited donor sample size. Therefore, a larger study involving multiple donors is required to confirm the generalized reproducibility of this approach.

## 3. Conclusions

In this study, we established a clinically aligned workflow for using mechanically minced human nasal septal cartilage (NSC) embedded in a collagen type I hydrogel as a potential one-step strategy for the treatment of focal cartilage defects. A brief antibiotic-based decontamination step reliably sterilized NSC biopsies without impairing chondrocyte viability or subsequent outgrowth, supporting the safe intra-articular use of NSC as a graft source. Minced NSC fragments in collagen I/PRP gels showed robust cell migration into the surrounding matrix and the formation of Safranin-O-positive, collagen II-rich cartilaginous tissue, confirming the overall feasibility of an Minced cartilage implantation-like approach using NSC.

Supplementation with Poloxamer 188 during mincing did not increase cell numbers or outgrowth but consistently improved histological tissue quality across donors. This was evidenced by more homogeneous proteoglycan deposition, higher Modified Bern Scores, and a more pronounced hyaline-like matrix phenotype. These effects may be attributed to the cytoprotective role of P188 in preserving chondrocytes from mechanical stress during fragmentation, thereby maintaining their biosynthetic capacity. Under identical culture conditions optimized for NSC, preliminary observations of osteoarthritic articular cartilage treated with P188 showed only limited outgrowth, fibroblast-like cell morphology, and minimal sGAG-rich matrix formation. These findings underscore the restricted regenerative potential of perilesional AC tissue typically used in clinical minced cartilage procedures and highlight the relative advantage of NSC as a superior tissue source.

Finally, we demonstrated the technical feasibility of generating a clinically relevant 1 mL MNC construct. This model maintained robust cell outgrowth and hyaline-like matrix islands, albeit with slightly lower overall histological quality compared to smaller-scale constructs. Taken together, our results identify NSC—particularly in combination with P188 and a collagen type I hydrogel—as a promising and potentially superior tissue source for MCI-based repair strategies.

This study presents several limitations. Firstly, the study lacks mechanistic validation of P188. While the membrane-stabilizing action of P188 is established in prior literature, this study did not include molecular or cellular assays confirming that P188 operates via the same mechanism in MNC. Future studies should address these limitations systematically to provide a more comprehensive understanding of P188 dose, exposure duration, and timing, in relation to its degree of protection. Another limitation of the study is the limited statistical power, which significantly impacts the interpretation of the observed performance of MNCs in the P188 group and the relatively modest outcomes in the case of MAC. Furthermore, the study lacks a true donor-, age-, sex-matched comparative evaluation of the performance of minced nasal cartilage and articular cartilage. Lastly, immunohistology assessments can be supplemented with molecular assays substantiating the role of key upstream markers. From the perspective of clinical translation, future studies should utilize suitable in vivo models to assess zonal tissue maturation and mechanical integration of the constructs. These findings can then be validated in larger and independent donor cohorts to fully establish the translational potential of this approach for the treatment of focal cartilage defects.

## 4. Materials and Methods

### 4.1. Collection of Human Cartilage Specimens

Human nasal and articular cartilage biopsies were provided by surgeons of the University Hospital of Basel. Samples were obtained from patients undergoing rhinoplasty surgeries or knee arthoplasty interventions, respectively. All patients provided their written informed consent before operation and harvesting, and their use occurred in accordance with the local ethical committee (University Hospital Basel).

### 4.2. Decontamination of Nasal Septal Cartilage Biopsies

Nasal septum cartilage (NSC) specimens (*N* = 5 patients) were briefly exposed to the patients’ own nasal mucosa (30 s) after collection to simulate contamination. Samples were subsequently transferred to the laboratory in transport medium (TM, see composition below) without antibiotics. Two 6 mm cartilage disks (ca 60–80 mg each) were harvested from each cartilage specimen: an untreated control (UT) and another subjected to the decontamination protocol described below (TR).

For the TR group, biopsies were immersed in 250 mL decontamination solution (DS) (see composition below) for 3 min under static conditions, followed by a 1 min dynamic wash (gentle manual swirling). This regimen was repeated in a second 250 mL DS bath. Subsequently, the biopsies underwent nine sequential washes in 45 mL DS aliquots (1 min each) under dynamic conditions. The UT group underwent an identical sequence using phosphate-buffered saline (PBS) without antibiotics. All washing steps were performed at room temperature under a Class II sterile hood.

Following the final wash, both NSC disks were transferred to antibiotic-free TM and stored overnight at 4 °C. The following day, each biopsy was rinsed twice in 45 mL PBS with a 1 min dynamic wash.

These decontamination steps were developed for practical application in the operating room immediately following tissue harvest. The procedure can be performed on a sterile side-table under laminar flow by a physician assistant or scrub nurse. Furthermore, this procedure does not extend the total surgical time, as it can be conducted concurrently with the surgical approach to the joint and the preparation of the cartilage lesion.

#### 4.2.1. Microbiological Testing

The following solutions were assayed for BacT/ALERT testing (bioMérieux, Marcy-l’Étoile, France) at the Department of Microbiology, University Hospital Basel: (i) TM before decontamination; (ii) PBS collected at the final step of decontamination. Aerobic and anaerobic bottles were incubated for up to 14 days as per the manufacturer’s instructions; positivity was determined by the instrument’s standard growth detection algorithms.

#### 4.2.2. Chondrocyte Isolation and Viability

Cartilage disks halves were enzymatically digested using Collagenase NB 4 Standard Grade (S17454): (SERVA Electrophoresis GmbH, Heidelberg, Germany). A digestion mixture of 7.5 mg collagenase in 5 mL of medium (complete medium for outgrowth (CM-O; composition below): DMEM, 1:1) was added to cartilage chips. Tubes were placed on a Solaris orbital shaker: (Thermo Fisher Scientific, Waltham, MA, USA) at 250–300 rpm, 37 °C for 22 h. The digest was filtered through a Falcon^®^ 100 µm sterile nylon cell strainer: (Corning Inc., Corning, NY, USA), collagenase was quenched with 20–30 mL CM–O, and cells were centrifuged (4 min, 1300 rpm, RT). Pellets were washed twice in CM–O prior to cells counted.

For viability, cells were stained with the LIVE/DEAD™ Viability/Cytotoxicity Kit (Thermo Fisher Scientific, Waltham, MA, USA) and analyzed by flow cytometry according to the manufacturer’s protocol. Events were recorded on a BD LSRFortessa Cell Analyser (Becton, Dickinson and Company, Franklin Lakes, NJ, USA). Gating was performed to exclude debris and doublets using FSC/SSC and area–height parameters. Viability was calculated as the percentage of live cells relative to the total cell population.

#### 4.2.3. Two-Dimensional Outgrowth Assay

The remaining halves of the NSC disks were minced into fragments smaller than 1 mm using a sterile No. 25 carbon-steel scalpel (REF 0212): (Swann-Morton, Sheffield, UK) in a rapid, repetitive chopping motion for 5 min. Fragments were divided into three equal portions and seeded into 24-well plates (92424): (TPP Techno Plastic Products AG, Trasadingen, Switzerland). Outgrowth cultures were performed in CM-O, a complete medium without antibiotics, to avoid confounding the decontamination readout. Cultures were maintained at 37 °C, 5% CO_2_. Medium changes were performed twice per week. At the study endpoint (confluence or day 30), the wells were harvested and digested overnight with proteinase K (1 mg mL^−1^ proteinase K in 50 mmol L^−1^ Tris with 1 mmol L^−1^ ethylenediaminetetraacetic acid, 1 mmol L^−1^ iodoacetamide and 10 mg mL^−1^ pepstatin A) for 16 h at 56 °C.

Total DNA was quantified using CyQUANT™ Cell Proliferation Assay (C35011): (Thermo Fisher Scientific, Waltham, MA, USA) against a 0–1000 ng mL^−1^ DNA standard curve. Cell numbers were estimated assuming 9.69 pg DNA per cell, an empirical average determined previously by cell counting and DNA quantification of enzymatically digested cartilage samples.

### 4.3. Cell Viability in Nasal Septal Cartilage After P188 Treatment

NSC biopsies from three male donors (mean age 26.7 ± 6.8 years) were placed on a sterile Teflon block and wetted with 50–100 µL of either Basic Medium (BM; Ctr) or Protection Medium (PM; BM supplemented with P188, composition detailed below) before being manually minced as described above. The resulting fragments were collected and transferred into tubes containing the respective medium (BM or PM) and incubated at 37 °C, 5% CO_2_ for 1 h. Thereafter, fragments from both conditions were transferred into tubes containing CM-V and cultured for an additional 24 h at 37 °C, 5% CO_2_. Subsequently, the NSC fragments were digested with type II collagenase, as previously detailed. The isolated cells were then mixed with Trypan blue and counted to assess cell viability, expressed as the percentage of live (unstained) cells relative to the total cell count.

### 4.4. Culture of MNC Generated Without or with P188 in Collagen Type I Gels

#### 4.4.1. Cartilage Mincing

NSC biopsies (*N* = 4; two male and two female donors; mean age 25.6 ± 6.0 years) and articular cartilage biopsies (*N* = 2; male donors; mean age 57.5 ± 0.7) were weighed. Tissue samples (60–80 mg) were wetted with 50–100 µL of either BM or PM and manually minced as described above. The resulting fragments were transferred into tubes containing fresh BM or PM and incubated at room temperature for approximately 30 min.

#### 4.4.2. Preparation of Platelet-Rich Plasma (PRP)

Peripheral venous blood from two healthy male donors (aged 27 years and 37 years) was processed with the ACP^®^ Double Syringe System (Arthrex, Naples, FL, USA) and ACD-A anticoagulant (Citra Labs, Braintree, MA, USA) according to the manufacturer’s instructions. Following centrifugation at 1500 rpm for 5 min at RT, 3–5 mL PRP were obtained from 15 mL whole blood, aliquoted, and stored at −80 °C (single freeze–thaw cycle) for up to 43 days. Non-activated PRP was used at 5% (*v*/*v*) in gels and chondrogenic medium. For the 0.5 mL constructs, PRP from a single donor (27-year-old) was used. To simulate a clinical scenario, the 1 mL constructs were generated from two distinct donors and supplemented with PRP derived from two different donors.

#### 4.4.3. Generation of Small (0.5 mL) and Large (1 mL) Scale Constructs

Neutralized collagen type I gels were prepared by mixing ChondroFiller® (NF250129FE): (meidrix Biomedicals GmbH, Esslingen Germany) and a neutralization solution (5GNL-MP240612): (meidrix Biomedicals GmbH, Esslingen, Germany) on ice (final pH ~7.4) to a final concentration of 8 mg mL^−1^ [41]. Minced cartilage suspensions were centrifuged (Heraeus Multifuge 3SR+ centrifuge): (Thermo Fisher Scientific, Waltham, MA, USA) (1400 rpm, 4 min, 20 °C); the supernatants were discarded, and the cartilage fragments were washed with PBS to remove residual fluid containing P188. Cartilage fragments and 5% (*v*/*v*) PRP were mixed into the neutralized collagen, dispensed, and allowed to gel for ≥ 30 min at room temperature (RT). Constructs were produced in two sizes as follows:

(i). Small scale 0.5 mL gel constructs were cultured in ultra-low attachment 24-well plates (174930): (Nunclon™ Sphera™, Thermo Fisher Scientific, Waltham, MA, USA) with 1.5 mL Chondrogenic Medium (ChM, see composition below) per well;
(ii). Large scale 1 mL gel constructs were cultured in Transwell^®^ 6-well inserts (3450): (Corning, NY, USA). For transwells, 1.6 mL ChM was placed inside the insert and 2.5 mL was placed in the well.

All constructs were cultured for up to 42 days in ChM at 37 °C, 5% CO_2_ with medium changed twice a week.

#### 4.4.4. Histology and Immunohistochemistry (IHC)

After 42 days, constructs were cut in halves; one half was fixed in 4% paraformaldehyde (24 h, at room temperature), paraffin-embedded, and sectioned at 5 µm on a Microm HM 355S rotary microtome (Thermo Fisher Scientific, Waltham, MA, USA). Sections from 6-9 levels per construct were collected. Sections collected on Polysine™ Adhesion Slides (J2800): (Epredia Netherlands B.V., Breda, Netherlands) were stained with Safranin-O/Fast Green and counterstained with hematoxylin (J.T. Baker, Radnor, PA, USA). Brightfield images were acquired on a Nikon Eclipse Ti2 microscope equipped with a Nikon DS-Ri2 color camera (Nikon Corporation, Tokio, Japan), using a CFI Plan Apo Lambda 20×/0.75 NA objective (Nikon Corporation, Tokio, Japan).

Adjacent sections collected on Histobond™+M (0811701): (Paul Marienfeld GmbH & Co. KG, Lauda-Königshofen, Germany) slides underwent automated IHC on Ventana DISCOVERY ULTRA (Roche Diagnostics, Basel, Switzerland). The primary antibodies used were as follows: collagen type II (mouse mAb, clone II-4C11 MA5-12789): Thermo Fisher Scientific, Waltham, MA, USA), Ki-67 (rabbit pAb, ab15580): (Abcam, Cambridge, UK), MMP14 (rabbit pAb, ab51074): (Abcam, Cambridge, UK), collagen type I (rabbit mAb, clone EPR7785, ab138492): (Abcam, Cambridge, UK). Isotype-matched mouse and rabbit IgG controls were processed in parallel.

#### 4.4.5. Image Analysis and Quantification

Analyses were performed in QuPath v0.5.0. [42] Images were color deconvolved in QuPath.

Tissue annotation: For col I and col II, a pixel thresholding approach was used, with pixel size 18 µm/pix, an average channel of red, green, and blue, and with gaussian filter and sigma 1. For Ki67 and MMP14 datasets, tissue was manually delineated. For collagen II-positive area, pixel classifier was trained to distinguish Col II-positive area from the gel area based on manual annotations. Classifier’s parameters: ANN_MLP, pixel size: 2.93 µm/pix; channels: red, green, blue; scales: 1,2; Qupath filters: Gaussian, Laplacian of Gaussian.

Cartilage regions have been manually delineated by an expert.

For collagen I-positive area, the pixel classifier was trained to distinguish the collagen I-positive area from the gel area based on manual annotations. Classifier’s parameters: ANN_MLP, pixel size: 0.73 µm/pix; channels: red, green, blue; scales: one; filters: Gaussian.

For Ki67 and MMP14-positive cells, cells were classified using a random tree-trained object classifier based on Ki67 +/− or MMP14 +/− annotations on DAB measurements, respectively.

#### 4.4.6. Outgrowth Score

Cell migration from native cartilage fragments into the hydrogel was assessed using a semi-quantitative scoring system (0–2), adapted from Levinson et al. [21]: 0 = no cells/only fragments; 1 = emergence at one–two sites without full colonization; 2 = cells distributed throughout the gel.

#### 4.4.7. Cell Colonization

Only nuclei outside native cartilage fragments were counted on Safranin-O/Fast Green sections using QuPath across all levels (six to nine per construct); the total number of cell nuclei per section was recorded. Cells in matrix were detected using QuPath’s watershed detection algorithm, using the following parameters: pixel size: 0.5 µm/pix; detection channel: optical density sum; threshold: 0.2; cellExpansionMicrons: 1 µm. Due to over-segmentation, cells were further classified into cells vs. non_cells using a trained OpenCVMLClassifier based on manual annotations and all calculated features. Cells in the cartilage area were segmented using Cellpose (v3.1.1) [43] with the following parameters: pixel size 0.37 µm/pix; cellprobThreshold: 0.0; flowThreshold: 0.4; model cyto2; diameter: 45, median filter: 2, within QuPath as an extension (v.0.9.2) (https://github.com/BIOP/qupath-extension-cellpose, accessed on 15 May 2024).

#### 4.4.8. Safranin-O-Positive Area

For the quantification of the Safranin-O-positive area, a supervised pixel classifier was implemented to segment red-stained regions from the surrounding hydrogel. The classifier’s parameters were as follows: ANN_MLP, pixel size: 2.93 µm/pix; channels: red, green, blue; scales: 1.0, 2.0; filters: Gaussian. The classifier was trained to distinguish between the hydrogel, newly formed sulfated glycosaminoglycans (sGAG), and native cartilage. For the final assessment of the total Safranin-O-positive area, comprising both newly formed sGAG and native cartilage, it was summed and expressed as a percentage of the total construct area.

#### 4.4.9. Modified Bern Score (MBS)

Safranin-O/Fast Green-stained sections were scored with the MBS (0–6) [19] which evaluates (i) Safranin-O-staining intensity (0 = none; 1 = weak; 2 = moderate and homogeneous; 3 = strong and homogeneous), and (ii) cell morphology (0 = condensed/pyknotic; 1 = spindle-shaped/fibrous; 2 = mixed spindle-shaped and rounded; 3 = predominantly rounded/chondrogenic). Each section was independently scored by three blinded operators, and the mean of the three scores was used as the final value for that section. Inter-operator reliability of the MBS was assessed using an interclass correlation coefficient (ICC). The calculated ICC was 0.879 (ICC (3,1), using two-way mixed-effects model, absolute agreement), indicating excellent reliability.

### 4.5. Media Composition

Basic medium (BM): High-glucose DMEM (10938-025): (Invitrogen, Waltham, MA, USA) supplemented with 10mM HEPES (Invitrogen, 15630-056), 1 mM sodium pyruvate (Invitrogen, 11360-039), 100 U mL^−1^ Penicillin, 100 µg mL^−1^ Streptomycin, and 0.29 mg mL^−1^ L-glutamine (Invitrogen, 10378-016).

Transport medium ™: High-glucose DMEM (Invitrogen, 10938-025) supplemented with 10 mM HEPES (Invitrogen, 15630-056), 1 mM sodium pyruvate (Invitrogen, 11360-039), and 2 mM GlutaMAX™ (Invitrogen, 35050061).

Decontamination solution (DS): PBS (Thermo Fisher, 10010023) containing 1% penicillin–streptomycin (15140122), 0.25 µg mL^−1^ amphotericin B, 10 µg mL^−1^ gentamicin (Gentamicin/Amphotericin R-015-10): (Invitrogen, Waltham, MA, USA).

Complete medium for outgrowth (CM-O): TM containing 5% fetal bovine serum (FBS 12491-015): (Gibco, Waltham, MA, USA).

Complete medium for viability (CM-V): BM containing 5% FBS.

Protection medium (PM): BM supplemented with 8 mg mL^−1^ Poloxamer 188 (Kolliphor^®^ P188); (BASF, Ludwigshafen, Germany) [23,24,33].

Chondrogenic medium (ChM): BM containing 5% (*v*/*v*) platelet-rich plasma (PRP), supplemented with 0.1 mM ascorbic acid-2-phosphate, and 10 mg mL^−1^ insulin (Actrapid^®^): (Novo Nordisk, Bagsværd, Denmark).

## Figures and Tables

**Table 1:** Transport media (TM) and media from untreated samples (UT, i.e., samples rinsed with only PBS) and antibiotic-treated biopsies (TR) (*N* = 5 donors) were routinely incubated for 14 days or until positivity (1–100 CFU per bottle) at 36 ± 1 °C using BacT/ALERT VirtuoTM automated culture system.

Result	TM	UT	TR
Positive	5/5	0/5	0/5
Negative	0/5	5/5	5/5

**Table 2:** Modified Bern Score sub-grading results of ctr and P188 constructs. Values are represented as Mean ± SD across *N* = 4 donors (6 constructs, 6–9 levels/construct). Donor-paired statistical analysis was performed using Wilcoxon matched-pairs signed-rank test, two-tailed.

	Intensity of Staining (Min 0, Max 3)	Cell Morphology (Min 0, Max 3)
Ctr	0.93 ± 0.66	1.66 ± 0.53
P188	1.24 ± 0.65	1.89 ± 0.54
*p* value	0.0012	0.0104

## Data Availability

The original contributions presented in this study are included in the article. Representative raw images used for creating pixel thresholders have been deposited in a repository accessible through the following link: 10.5281/zenodo.18269241.

## Supplementary Materials

The following supporting information can be downloaded at https://www.mdpi.com/article/10.3390/gels12030190/s1; Table S1: Aerobic and anaerobic culture results and time-to-positivity of transport medium (TM); Figure S1: Effect of Poloxamer 188 on nasal chondrocyte viability after cartilage mincing; Figure S2: Safranin-O/Fast Green-staining images of “worst” donor-paired outcomes for control and P188 groups; Figure S3: Isotype controls.

## Abbreviations

The following abbreviations are used in this manuscript:

Ab	Antibody		
ACD	Anticoagulant Citrate Dextrose Solution		
AC	Articular Cartilage		
ACI	Autologous Chondrocyte Implantation		
BM	Basic Medium		
ChM	Chondrogenic Medium		
CM	Complete Medium		
CM-O	Complete medium for Outgrowth		
CM-V	Complete Medium for Viability		
Col I	Type I collagen		
Col II	Type II collagen		
Ctr	Control		
DMEM	Dulbecco’s Modified Eagle Medium		
DNA	Deoxyribonucleic Acid		
DS	Decontamination Solution		
FBS	Fetal Bovine Serum		
FSC	Forward Scatter		
GAG	Glycosaminoglycan		
HEPES	4-(2-hydroxyethyl)-1piperazineethanesulfonic acid		
IHC	Immuno(H)isto(C)hemistry		
MAC	Minced Articular Cartilage		
MBS	Modified Bern Score		
MCI	Minced Cartilage Implantation		
MNC	Minced Nasal septal Cartilage		
MMP14	Matrix Metalloproteinase-14		
MT1MMP	Membrane Type 1 Matrix Metalloproteinase		
NCs	Nasal Chondrocyte		
NSC	Nasal Septal Cartilage		
OA	Osteoarthritis		
P188	Poloxamer 188		
PBS	Phosphate-Buffered Saline		
PM	Protection Medium		
PRP	Platelet-Rich Plasma		
sGAG	Sulfated Glycosaminoglycan		
SSC	Side Scatter		
Rpm	Revolution per minute		
RT	Room Temperature		
TM	Transport Medium		
TR	Treated		
SD	Standard Deviation		
UT	Untreated		
