# Supplementary material for: Migration and Chondrogenesis of Cells from Minced Nasal Cartilage in Type I Collagen Hydrogel: A Workflow for One-Step Engineering of Injectable Grafts
Source: Gels. 2026 Feb 25;12(3):190. doi: 10.3390/gels12030190 (PMC13025651; doi:10.3390/gels12030190)
Supplement: Supplementary file 1 [file gels-12-00190-s001.zip › gels-4057198-supplementary.pdf]

Supplementary Materials

Table S1 Aerobic and anaerobic culture results and time-to-positivity of Transport Medium (TM). (*N* = 5 donors). Samples were routinely incubated for 14 days or until positivity (1-100 CFU per bottle) at 36±1°C using the BacT/ALERT Virtuo™ automated culture system. Time to positivity (TTP) and identified bacterial species are shown for both culture types.

	Aerobic		Anaerobic	
Donor	Time-to-positivity	Species	Time-to-positivity	Species
1	1d 8h 54min	*Staphylococcus epidermidis*	1d 12h 48min	*Staphylococcus epidermidis*
2	n.a.	n.a.	15h 36min	*Staphylococcus aureus*
3	< 24h	*Staphylococcus epidermidis*	< 24h	*Staphylococcus epidermidis*
4	1d 10h 18min	*Staphylococcus epidermidis*	n.a.	n.a.
5	12h 30min	*Staphylococcus aureus*	17h 36min	*Staphylococcus aureus*

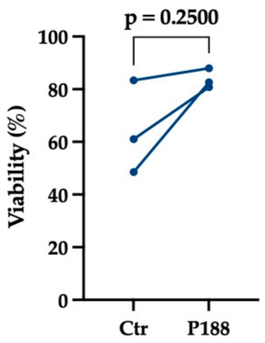

Figure S1 Effect of Poloxamer 188 on nasal chondrocyte viability after cartilage mincing. Nasal septal cartilage samples from three male donors were minced and incubated for 1 h in basic medium (Ctr) or protection medium containing P188, followed by 24 h of culture in basic medium containing 5% FBS. Viability of released cells was assessed by Trypan blue exclusion (by three different operators). Donor-paired quantitative analysis (*N* = 3 donors, Wilcoxon matched-pairs signed rank test, two-tailed), Viability ($p = 0.2500$), Mean±SD: Ctr = 64.3 ± 17.7, P188 = 83.8 ± 3.7.

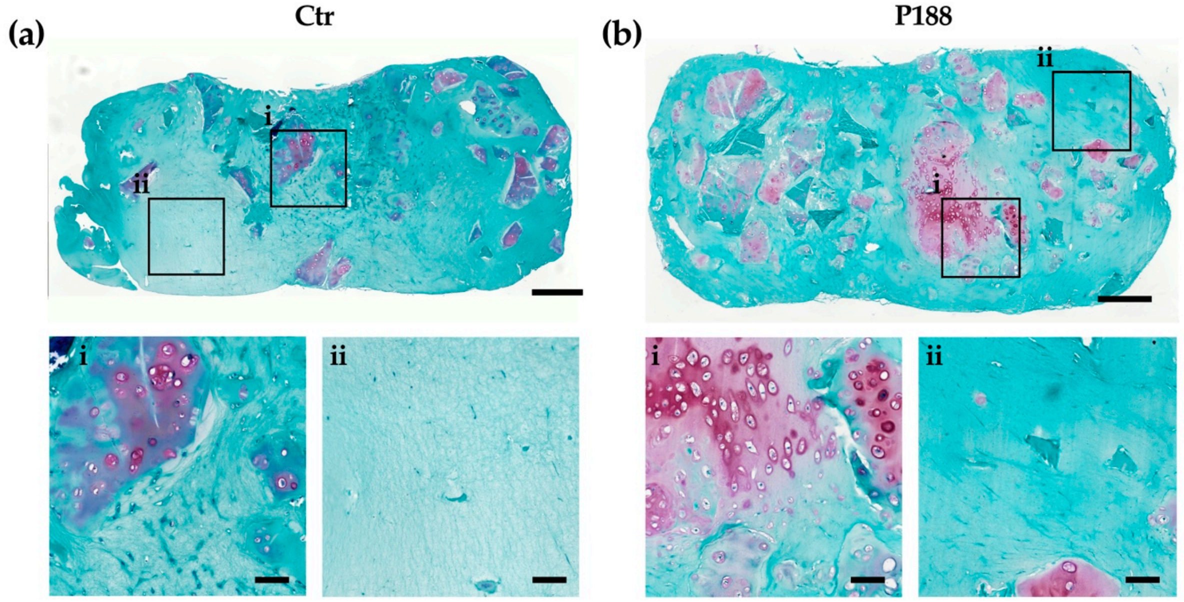

Figure S1. Safranin-O/Fast Green-staining images of "worst" donor-paired outcomes for control and P188 groups, **(a)** and P188 **(b)** groups. Scale bars: overviews 500 µm, zooms 100 µm.

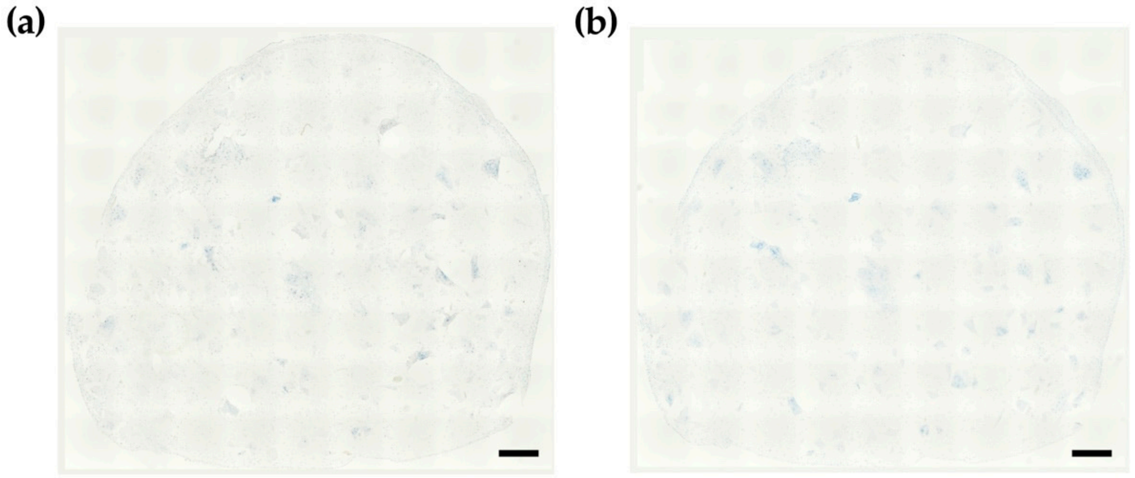

Figure S3 Isotype controls. Representative sections of cartilage constructs stained with **(a)** mouse IgG and **(b)** rabbit IgG isotype control antibodies, processed in parallel with the primary antibodies (collagen type II, collagen type I, Ki-67, MMP14). Scale bar: 500 µm.
